# Clinical Frailty Scale predicts overall survival after colon cancer surgery in people aged 80 years and older: A prospective multicentre observational study

**DOI:** 10.1111/codi.70190

**Published:** 2025-08-05

**Authors:** Susanna Niemeläinen, Marja Hyöty, Anu Ehrlich, Esa Jämsen, Selja Koskensalo, Jyrki Kössi, Tarja Pinta, Hanna Vihervaara, Heini Huhtala

**Affiliations:** ^1^ Department of Gastroenterology and Alimentary Tract Surgery Tampere University Hospital Tampere Finland; ^2^ Department of Abdominal Surgery Helsinki University Hospital Helsinki Finland; ^3^ Faculty of Medicine Helsinki University Helsinki Finland; ^4^ Department of Geriatrics Helsinki University Hospital Helsinki Finland; ^5^ Department of Surgery Päijät‐Häme Central Hospital Lahti Finland; ^6^ Seinäjoki Central Hospital Seinäjoki Finland; ^7^ Division of Digestive Surgery and Urology Turku University Hospital Turku Finland; ^8^ Faculty of Medicine Turku University Turku Finland; ^9^ Faculty of Social Sciences Tampere University Tampere Finland

**Keywords:** age patients, Clinical Frailty Scale, colon cancer, overall survival, surgery

## Abstract

**Aim:**

An increasing number of elective colon cancer surgeries are performed on older patients. Frail patients are at a higher risk of postoperative adverse events and early mortality. This prospective, multicentre, observational study aimed to analyse preoperative screening tools and the long‐term survival of older patients having elective colon cancer surgery. The focus was on patients who survived more than 3 months postoperatively, excluding the effect of early postoperative mortality.

**Methods:**

Patients aged ≥80 years with electively operated stage I‐III colon cancer were recruited. Prospectively collected data included comorbidities, functional and frailty status, postoperative outcomesand long‐term survival. Univariate and multivariable Cox regression analyses were conducted to determine factors associated with long‐term survival.

**Results:**

A total of 227 surgical patients were included. Survival rates at 1 and 3 years were 94% and 74% for all patients and 86% and 57% for patients with Clinical Frailty Scale (CFS) 5–9, respectively. In multivariable regression analysis, CFS 5–9 (HR 3.16, 95% CI 1.31–7.63, *p* = 0.011) and tumour stage III (HR 2.50, 1.16–5.39, *p* = 0.020) were the patient‐related variables affecting survival among those surviving over 3 months postoperatively.

**Conclusions:**

Preoperative frailty assessed by CFS predicts long‐term survival in older patients after curative colon cancer surgery.

**What does this paper add to the literature?**

This study showed that frail patients have significantly worse overall survival after curative colon cancer surgery. As a predictor of long‐term survival, the Clinical Frailty Scale might be a useful tool in helping treatment decision‐making.

## INTRODUCTION

In Finland, the number of individuals aged 80 years or older is expected to increase 70% by the year 2045 [1]. The risk of developing colon cancer increases with age. Thus, colon cancer is increasingly diagnosed in older patients, being already the third most common cancer in Finland and the most common for both genders over the age of 80 years [2]. These changing demographics have a profound impact on cancer care as increasing numbers of invasive surgical treatments are required [3].

The primary treatment of colon cancer is surgery with curative intent [4, 5]. Current studies present comparable disease‐specific long‐term survival rates for all age groups, advocating invasive treatment [6, 7]. On the contrary, older patients are at higher risk of experiencing postoperative complications and even mortality after surgical treatment [8, 9], and have worse overall long‐term survival compared to younger patients due to competing causes of death [6, 7, 10]. Older individuals constitute a heterogeneous group of patients with a wide range of comorbidities, disability, and frailty, all of which are strongly associated with reduced short‐ and long‐term survival after surgery [11, 12]. Consequently, decision‐making concerning radical surgery on older patients might be challenging as treatment should focus not only on survival data but also on functional and quality of life outcomes [13, 14].

Clinicians need to emphasise the risk assessment of comorbidities, functional and frailty status in older colon cancer patients. Unfortunately, preoperative comprehensive geriatric assessment of colon cancer patients is often time‐consuming and requires specially trained geriatricians [15, 16]. The age‐adjusted CCI (AA‐CCI) score, which evaluates the cumulative burden of comorbidities, helps to identify aged patients at greater risk of short‐ and long‐term mortality [17]. Population‐based studies from Finland reported that higher AA‐CCI was an independent prognostic factor for the 5‐year overall survival (OS) of colon cancer patients operated during 2000–2016 [10, 18].

Clinical Frailty Scale (CFS) has been developed for rapid frailty screening without the need for specific geriatric expertise or functional testing [19]. CFS has been widely used, and it correlates well with postoperative complications and with functional outcomes [8, 20]. A retrospective population‐based study from Japan showed that preoperative frailty (CFS ≥ 4) was independently associated with shorter disease‐free and overall survival in a 4.5‐year follow‐up with colorectal cancer patients [21]. Otherwise, an association of CFS‐based frailty and overall survival from cancer surgery remains unclear due to a lack of studies in very old patients.

In our previous study on colon cancer patients, preoperative frailty status assessed by CFS was a major risk factor for postoperative complications [8]. This study aimed to analyse long‐term survival in the same cohort. Another aim was to assess preoperative screening tools such as AA‐CCI [17], onco‐geriatric screening tool G8 [22] and CFS and their predictive value for long‐term survival. To exclude the effect of early postoperative mortality, this study especially focused on patients who survived at least 3 months after surgery.

## PATIENTS AND METHODS

### Study design

A multicentre, prospective observational cohort study of patients aged 80 years or older with Stage I–III colon cancer was conducted in nine Finnish public hospitals. Recruitment started on 1 April 2019, and continued until 31 March 2021. In Finland, the standardised approach to diagnosing and treating colon cancer has been established by recently published national guidelines [5]. The study hospitals cover 70% of Finland's population, thus providing a current nationwide spectrum of postoperative outcomes for older colon cancer patients.

This study followed the STROBE guidelines [23]. The Ethics Committee of Tampere University Hospital (reference approval number R19028) and the institutional review boards at each study site approved the study protocol. The study was registered in ClinicalTrials.gov (NCT03904121) in April 2019. Our previously published articles have described the detailed study protocol, data collection and short‐term results [8, 24].

### Participants and data collection

All recently diagnosed colon cancer patients aged 80 years or over referred to the study hospitals for operative treatment were eligible to participate in the study. Patients who survived less than 3 months after surgery, had metastatic disease, or emergency operations were excluded. In addition, patients who consented to the study but were treated non‐operatively or were found to have metastatic or benign disease at surgery were excluded from the present analysis.

Final data, including dates and causes of death, was completed from hospital patient records and from patient questionnaires filled by mail or phone interview by surgeons in study hospitals. The data was collected and managed using the electronic web‐based software platform REDCAP (Research Electronic Data Capture) database [25].

Frailty was determined by the Clinical Frailty Scale (CFS) version 1.0 assessed by surgeons at the study hospitals [19]. For this study, the preoperative frailty was divided into four groups according to CFS status in order to analyse OS with different frailty stages separately. Based on CFS, the patients were categorised as fit (CFS 1–2), managing well (CFS 3), pre‐frail (CFS 4) or frail (CFS 5–9).

Other patient‐ and surgery‐related variables analysed in this study were age, type of living, activities of daily living (ADL), mobility, hospital admissions 6 months before surgery, number of medications, comorbidities, AA‐CCI [17], American Society of Anaesthesiologists physical status classification ASA [26], onco‐geriatric screening tool G8 [22], Mini Nutritional Assessment‐Short Form (MNA‐SF) [27], preoperative haemoglobin (g/L), albumin (g/L), estimated glomerular filtration rate GFR (mL/min), postoperative outcomes, tumour stage according to the Union for International Cancer Control (UICC) TNM classification [28].

### Outcomes

Data analysis focused on those patients who survived over 3 months after the primary cancer operation, intending to exclude the effect of early postoperative mortality. The primary outcome measure was overall survival (OS), which was calculated from the date of surgery to the date of death from any cause or to the closure of follow‐up on 31 March 2024. OS was reported for all patients surviving 3 months after surgery and separately for the four CFS subgroups.

### Statistical analysis

Demographic data and outcomes were reported as percentages. Associations between the categorical variables were tested with the chi‐square test or the Fisher exact test, when appropriate. The Kaplan–Meier method was used to describe the OS distributions with the log‐rank test. Log minus log plots were used to validate the Cox proportional hazard assumption.

A univariate and multivariable analysis of the factors predicting OS were carried out using Cox's regression model. Variables that were clinically (ASA, AA‐CCI, CFS, G8) and statistically significant (*p* < 0.05) in the univariate model were included in the multivariable model. Variables (age, activities of daily living (ADL), mobility, MNA‐SF) that intercorrelated with other included variables (CFS, G8) or postoperative variables (operation type, adverse events) were excluded from the multivariable model. Statistical analyses were performed using SPSS version 26 (IBM, Armonk, New York, USA).

## RESULTS

### Patients and clinical characteristics

**FIGURE 1:**
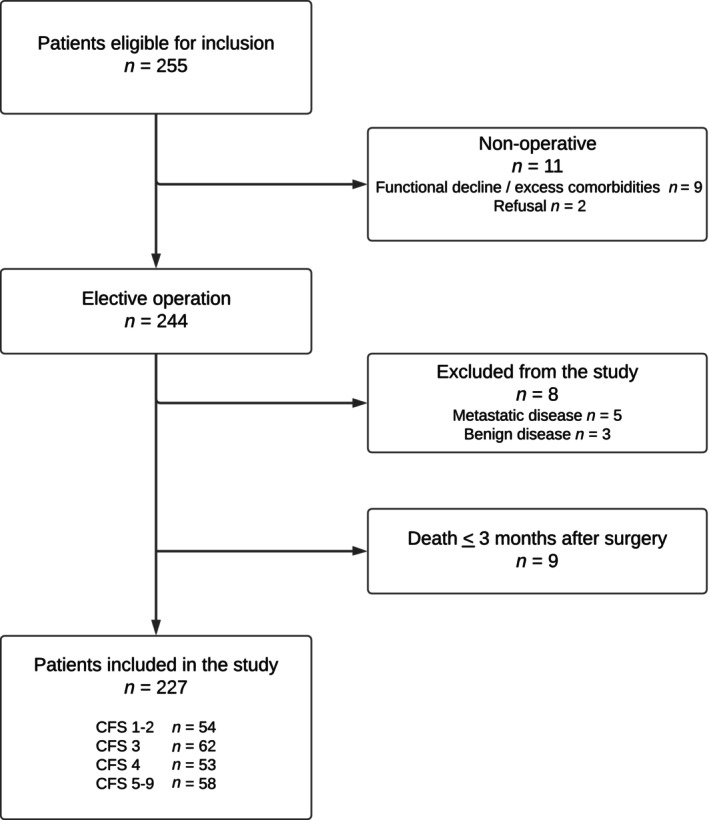
Patients flowchart.

Of the 257 eligible patients, 244 (96%) were operated on. Eight patients were excluded from this study because of benign or metastatic disease, and nine patients were excluded during the first 3 months (Figure 1). The overall 30‐day and 90‐day mortality was 1.7% (4/236) and 3.8% (9/236), respectively.

The final study cohort consisted of 227 patients. Their median age was 84 years (range 80–97 years), and 59% were women. Most patients had a CCI score ≤ 6 (62%), were classified as ASA 3 (67%) and scored ≤ 14 (90%) in the G8. According to the CFS, 54 (24%) patients were Grade 1–2, 62 (27%) were Grade 3, 53 (23%) were Grade 4 and 58 (26%) were Grade 5–9, respectively. Thus, 49% (111/227) of patients were considered as prefrail or frail (CFS ≥ 4).

Most of the surgeries were performed for right‐sided cancer (70%) and laparoscopically (72%). Postoperative complications were recorded in 88 patients (39%), with 48 (21%) patients having surgical and 44 (19%) non‐surgical complications. In frail patients (CFS 5–9), the rates were 50%, 22% and 33%, respectively. According to the Clavien‐Dindo classification, 24 (11%) patients had severe complications (Grade III–IV). Reoperation was needed in 18 patients (7.9%). Ninety‐three patients (41%) were discharged to other medical facilities, such as wards in primary health care. Within 30 days of discharge, readmission occurred in 21 patients (9.3%).

According to the Union for International Cancer Control stages, TNM stages were as follows: Stage I, 53 patients (23%); Stage II, 113 patients (50%) and Stage III, 61 patients (27%). Postoperative adjuvant therapy was given to 10% of all patients and 30% of Stage III patients. The recurrence rate was 7.0% (available for 219 patients) involving metastasis in the liver (4 patients), lung (5 patients), peritoneum (2 patients) and other locations (3 patients). Recurrences developed in 1.9%, 2.7% and 19.7% of tumour stage I, II and III, respectively.

The baseline and clinical characteristics of the patients who survived over 3 months after surgery are shown in Table 1. The population is presented in four different CFS groups in Supplement 1.

**TABLE 1:** Baseline and clinical characteristics with patients, who survived at least 3 months after surgery.

	*n*/median	%/(range)
Sex ratio (female /male)	134/93	59.0/41.0
Age, years	84.3	(80–97)
80–84	128	56.4
85–89	67	29.5
≥90	32	14.1
BMI, kg/m^2^	25.7	(16.5–40.0)
<24	75	33.0
24–29	102	45.0
>29	50	22.0
Living status		
Home	223	98.2
Nursing home	4	1.8
Need for support with activities of daily living		
Independent	121	53.3
Outdoors independent	30	13.2
Out and indoors with housework	50	22.0
Out and indoors with basic activities	26	11.5
Mobility outdoors		
Outdoors unassisted	163	71.8
Outdoors assisted	56	24.7
No outdoor activity	8	3.5
Mobility		
Independent	138	60.8
Independent with walking aid	81	35.7
Dependent of support care or unable to move	8	3.5
Use of walking aid	105	46.5
Hospital admissions ≤6 months		
No	117	51.5
One or more	110	48.5
Number of medications		
<5	86	37.9
≥5	141	62.1
Comorbidities		
Hypertension	160	70.5
Cardiovascular disease^a^	123	54.2
Diabetes	72	31.7
Renal disease	40	18.3
Cerebrovascular disease	29	12.8
Pulmonary disease	28	12.3
History of cognitive impairment	71	31.3
Clinical Frailty Scale CFS (1–9)		
1–2	54	23.7
3	62	27.3
4	53	23.4
5–9	58	25.6
G8 score (0–17)		
<12	105	46.3
12–14	100	44.1
>14	22	9.6
Charlson Comorbidity Index CCI (4–15)		
4–6	141	62.1
≥7	86	37.9
ASA score (2–4)		
2	59	26.0
3	152	67.0
4	16	7.0
Mini Nutritional Assessment‐Short Form (0–14)		
0–7 (malnutrition)	49	21.6
8–11 (risk of malnutrition)	153	67.4
≥12 (normal nutrition)	25	11.0
Haemoglobin (g/L)		
≤ 120	150	66.1
> 120	77	33.9
Albumin (g/L) (missing 19)		
≤30	38	18.3
31–34	67	32.2
>34	103	49.5
Estimated glomerular filtration rate GFR (mL/min)		
<45	45	19.8
45–60	56	24.7
>60	126	55.5
Procedure		
Right‐sided colectomy	158	69.6
Left‐sided colectomy	65	28,6
Another colonic resection	4	1.8
Type of surgery		
Open	42	18.5
Laparoscopy	164	72.2
Conversion	21	9.3
Postoperative complications	88	38.8
Surgical complications	48	21.1
Non‐surgical complications	44	19.4
Clavien‐Dindo classification		
0	139	61.2
I–II	64	28.2
III–IV	24	10.6
Length of hospitalisation (days)	5.0	1–36
Reoperation	18	7.9
Readmission	21	9.3
Discharge destination from hospital		
Home	134	59.0
Other medical facilities	93	41.4
TNM stage		
I	53	23.3
II	113	49.8
III	61	26.9
Postoperative adjuvant therapy	22	9.8

^a^ Coronary artery disease + congestive heart failure + peripheral artery disease + arrhythmia.

### Long‐term survival

**FIGURE 2:**
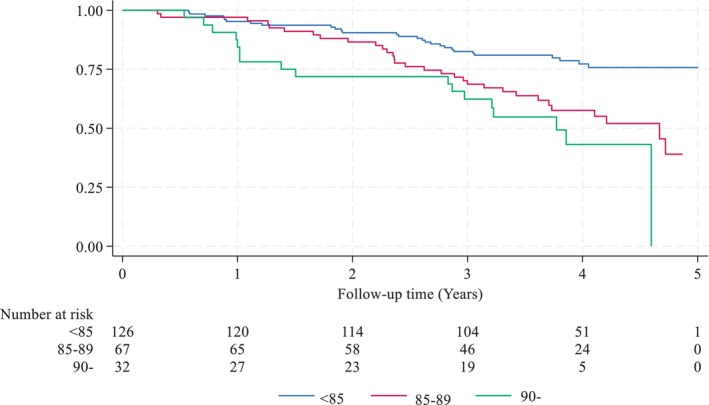
Kaplan–Meier analysis of age‐related overall survival in patients who survived at least 3 months after surgery (*p* < 0.001, log‐rank).

Of the whole study cohort, a total of 78 patients (34%) died during the follow‐up period (median follow‐up of 4.0 years, ranging from 91 days to 5 years). Therefore, the OS was 66%. The 1‐ and 3‐year survival rates were 94% (214/227) and 74% (169/227), respectively. The corresponding numbers were 95% and 83% for ages 80–84 years, 97% and 67% for ages 85–89 years and 84% and 59% for 90 years and over, respectively (log‐rank *p* < 0.001; Figure 2).

The median survival times with tumour stages I, II and III were 4.7 (95% CI 4.5–4.8), 4.9 (4.8–5.0) and 4.0 years (3.6–4.4), respectively (log‐rank < 0.001).

**FIGURE 3:**
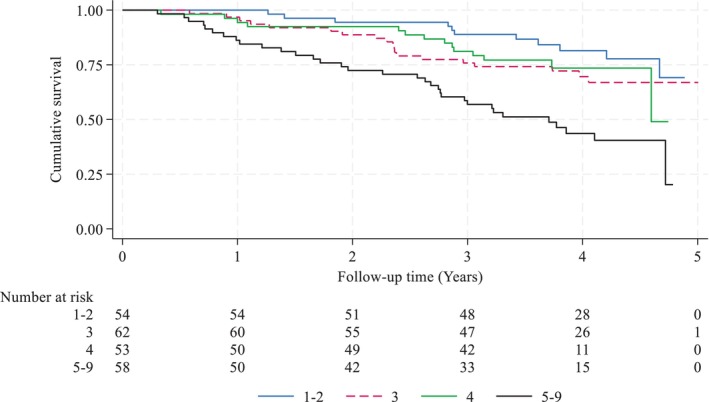
Kaplan–Meier analysis of Clinical Frailty Scale (CFS)‐related overall survival in patients who survived at least 3 months after surgery (*p* = 0.005, log‐rank).

For the different CFS groups the 1‐year, 3‐year and total follow‐up survival were 100%, 89% and 80% (CFS 1–2), 97%, 76% and 69% (CFS 3), 94%, 77% and 72% (CFS 4) and 86%, 57% and 43% (CFS 5–9), respectively (log‐rank < 0.001; Figure 3). With CFS 7–8, the respective numbers were 83%, 17% and 0%. The median survival time for CFS 5–9 was 3.7 years (CI 95% 3.0–4.5). With CFS 7–8, the median survival time of 2.3 years (CI 95% 1.4–3.1) compared to 3.7 years (95% CI 3.1–4.4) for patients with CFS 5–6.

The most common causes of death (available for 57 patients) were cardiopulmonary diseases (32%), colon cancer (26%), acute injury such as hip fracture (16%) and dementia (12%).

### Factors influencing long‐term survival

Univariate Cox regression analysis showed that older age, the need for support with ADL and mobility assistance, multiple hospital admissions <6 months before surgery, chronic renal insufficiency (GFR < 45 mL/min), G8 < 8, CFS ≥ 5, ASA > 3, MNA‐SF < 8, haemoglobin < 120 g/L and albumin level < 31 g/L were associated with poor OS. Of other factors, tumour stage III, open surgery, non‐surgical and severe complications were associated with worse OS (Supplement 2).

Multivariable regression analysis showed that CFS 5–9 (HR 3.16, 95% CI 1.31–7.63, *p* = 0.011) and tumour stage III (2.50, 1.16–5.39, *p* = 0.020) were associated with diminished overall survival (Table 2). When only preoperative screening factors (ASA, AA‐CCI, CFS, G8, haemoglobin, albumin and GFR) were analysed in multivariable regression analysis, CFS ≥ 5 (2.94, 1.23–7.03, *p* = 0.015), albumin level < 31 g/L (1.97, 1.03–3.74, *p* = 0.039) and GFR < 45 mL/min (2.02, 1.07–3.82, *p* = 0.030) were associated with reduced OS (Supplement 3).

**TABLE 2:** Selected factors influencing survival with patients who survived at least 3 months after colon cancer surgery (Cox regression analysis).

	Univariate			Multivariable		
	HR	95% CI	*p*‐value	HR	95% CI	*p*‐value
ASA						
2	1			1		
3	1.40	0.80–2.46	0.244	0.82	0.39–1.73	0.609
4	2.94	1.36–6.36	**0.006**	1.37	0.44–4.27	0.584
AA‐CCI						
4–6	1			1		
>6	1.38	0.89–2.17	0.155	0.96	0.83–1.11	0.614
CFS						
1–2	1			1		
3	1.73	0.82–3.63	0.150	1.44	0.61–3.40	0.403
4	1.73	0.79–3.80	0.167	1.54	0.61–3.91	0.366
5–9	4.05	2.04–8.04	**<0.001**	3.16	1.31–7.63	**0.011**
G8 score						
0–11	3.83	1.19–12.3	**0.024**	1.59	0.46–5.46	0.462
12–14	2.31	0.70–7.57	0.167	1.21	0.35–4.25	0.765
>14	1			1		
Haemoglobin (g/L)						
≤120	2.42	1.40–4.20	**0.002**	1.48	0.78–2.79	0.229
>120	1			1		
Albumin (g/L)						
<31	2.98	1.68–5.29	**<0.001**	1.84	0.95–3.58	0.072
31–34	1.29	0.74–2.27	0.375	0.96	0.52–1.77	0.895
>34	1			1		
GFR (mL/min)						
<45	2.11	1.23–3.61	**0.006**	1.60	0.80–3.18	0.184
45–60	1.63	0.96–2.80	0.073	1.54	0.82–2.88	0.177
>60				1		
Hospital admissions <6 months						
None				1		
One or more				1.70	0.99–2.91	0.054
Tumour stage						
1	1			1		
2	1.33	0.67–2.63	0.419	1.20	0.56–2.53	0.643
3	3.41	1.73–6.74	**<0.001**	2.50	1.16–5.39	**0.020**

Abbreviations: AA‐CCI, Age‐Adjusted Charlson Comorbidity Index; ASA, American Society of Anaesthesiologists; CFS, Clinical Frailty Scale; GFR, estimated glomerular filtration rate.

## DISCUSSION

This prospective, nationally representable multicentre study showed that patients with CFS 5–9 showed significantly poorer long‐term OS, emphasising the value of preoperative frailty screening. We call for shared decision‐making and aim for individualised colon cancer treatment.

Postoperative complications are a major risk factor for short‐term mortality for older patients [29]. We reported 30‐day and 90‐day mortality rates of 1.7% and 3.8%, respectively. The results verified a remarkable reduction from previously reported registry‐based studies [6, 30]. These improvements in short‐term postoperative outcomes are a result of preoperative risk assessments, successful prehabilitation, and enhanced perioperative management used in all study hospitals [31, 32]. Altogether, this development has shifted focus to longer‐term outcomes, assessed in this study.

A recently published study from Finland reported that patients between 80 and 84 years of age with localised or locally advanced colon cancer (Stages I–III) had 5‐year OS ranging from 37% to 59%. For patients over 90 years, OS diminished to 12%–32% [6]. Another registry‐based study of older patients from Australia reported similar 5‐year OS of 57% and 24% [33], and a population‐based Dutch study with over 6500 colon cancer patients over 80 years showed a 3‐year OS of 67% [7]. Our study demonstrated good results for operative treatment of colon cancer since the 3‐year OS was 74% for all patients and exceeded 50% also among those aged over 90. Life expectancy in Finland has increased remarkably, and in 2025 was 10.2 and 8.4 years for female and male individuals, respectively, over 80 years of age. For females and males over 90 years, the respective numbers were 4.5 and 3.8 years [1]. Thus, considering the very poor results of non‐operative treatment and the high life expectancy of octogenarians, a patient's chronological age alone should not result in undertreatment of fit older patients.

Comorbidities are frequent in older colon cancer patients, and prevalence rates of 30%–40% with at least one chronic condition are documented [34]. Comorbidities increase the risk of mortality both in the general population and cancer patients. A meta‐analysis of 37 cohort studies of colorectal cancer demonstrated that patients with moderate and severe comorbidities had a significantly increased risk of 30‐day mortality, as well as long‐term overall and cancer‐specific mortality [11]. Comparable results were verified in two population‐based studies from Finland [10, 18]. In our study, 38% of patients had an AA‐CCI score > 6, but a higher value did not significantly predict lower OS when other patient‐related factors were considered. This suggests that preoperative risk assessment with comorbidity optimisation has been adopted as an everyday practice in the study hospitals.

Frailty is a state of vulnerability, characterised by an age‐associated decline in physiological reserve and function across multiple organ systems [35]. However, not all older patients are frail. A recently published meta‐analysis demonstrated prevalence rates of 19%–55% of frailty in patients undergoing colorectal cancer surgery [36]. Frailty is strongly associated with postoperative short‐term adverse events [8, 37] as well as poor long‐term survival, prolonged disability and poor functional recovery in colorectal cancer surgery [13, 38]. Preoperative frailty documentation is highly advised by national surgical guidelines in the United Kingdom, United States and Finland [5, 39, 40]. Our study extends the results of a previous meta‐analysis by demonstrating significantly worse OS in frail compared to fit patients [38]. In our study, 26% of patients were frail (CFS 5–9), and their 1‐ and 3‐year OS was 86% and 56% compared with CFS 1–2 patients' OS 100% and 89%, respectively. Of note, metastasised cancer was more common in patients with CFS 3 than with CFS 4, possibly explaining worse OS in CFS 3 patients. Thus, the optimisation of comorbidities and nutritional status combined with comprehensive geriatric assessment might have a positive impact on patients' long‐term survival [41]. There was no structured prehabilitation programme in the study hospitals [42]. Prehabilitation of frail patients may optimise postoperative results, but more information is needed [43]. Some of our study hospitals have already instigated prehabilitation programmes after this study.

Preoperative frailty screening tools are needed to identify patients at risk of postoperative adverse events and poor long‐term survival. Ideally, these screening tools could help surgeons to identify patients who need preoperative comprehensive geriatric assessment [15, 16]. Frailty instruments should be easy to implement and reliably predict adverse outcomes of surgery. CFS is a well‐validated, clinical judgement‐based tool for rapid frailty screening [19]. In our study, we identified similarities between CFS scores and other geriatric syndromes associated with frailty (Supplement 2), which supports the feasibility and usefulness of the CFS in older colon cancer patients. Our previous study showed a significant association between CFS and postoperative complications [8]. In this study, we demonstrated that CFS 5–9 was the only preoperative screening factor with tumour stage III (*p* = 0.020), which was associated with diminished OS in the multivariable analyses (*p* = 0.011). After 3 years of surgery, only 17% of CFS 7 patients were alive, supporting previous findings. There is no present consensus on the most clinically useful tool to assess frailty as a predictor of postoperative adverse events or long‐term survival [44]. The current study, though, indicates that CFS assessment can be integrated into surgical units to improve patients' postoperative risk assessment. Consequently, further prospective observational studies are needed to examine the association between preoperative frailty and patient survival in colon cancer treatment.

When planning colon cancer treatment for older patients, the goals of care should be discussed with the patient and their family members, as well as with a colon cancer multidisciplinary team. Together with earlier studies [8, 9, 10, 21], this study gives information about real‐life outcomes in terms of the predictive risks of complications, long‐term survival and decline in functional status. Older patients may value their functional status more than invasive cancer treatments. Good quality of life and maintaining independence are essential outcomes for older patients [13, 14].

Although an observational study cannot conclude the advantage of surgery, it is not realistic to perform a randomised trial in this older patient group. Instead, it is clinically more reasonable to study outcomes in an observational setting with more relevance to real‐life settings. The strength of this study is that it included only patients 80 years and older, which is the fastest growing subgroup of the Finnish population. In addition, all patients were operated on electively with an opportunity to perform a cautious preoperative risk assessment and to achieve curative‐intent surgery. Consequently, this study gives a representative, nationwide picture of current cancer treatment and long‐term survival data concerning older patients' colon cancer surgery.

There are some limitations to this study. It was recognised that the tools used (AA‐CCI, G‐8, CFS and MNA‐SF) represented screening tests, and some of them were surgeon‐dependent; hereby, geriatric evaluation would be needed for precise diagnosis of frailty and other geriatric syndromes. We could not identify all causes of death from hospital patient data. Thus, the exact number of cancer recurrences and cancer‐related deaths was incomplete. Since functional status, independence and quality of life are highly valued by older people, future studies on this multicentre data will focus on long‐term functional recovery.

In conclusion, this study extends our earlier findings from the same cohort [8] and suggests that frail patients have significantly worse overall survival after elective, curatively aimed colon cancer surgery. The use of CFS in preoperative risk assessment as a tool for predicting long‐term survival might be useful and helpful in treatment decision‐making.

## AUTHOR CONTRIBUTIONS

**Susanna Niemeläinen:** Conceptualization; methodology; resources; data curation; investigation; validation; formal analysis; project administration; writing – original draft; writing – review and editing; funding acquisition; supervision. **Marja Hyöty:** Conceptualization; methodology; writing – review and editing. **Anu Ehrlich:** Data curation; investigation; writing – review and editing. **Esa Jämsen:** Conceptualization; methodology; writing – review and editing. **Selja Koskensalo:** Data curation; investigation; writing – review and editing. **Jyrki Kössi:** Conceptualization; methodology; writing – review and editing; data curation; investigation. **Tarja Pinta:** Data curation; investigation; writing – review and editing. **Hanna Vihervaara:** Data curation; writing – review and editing; investigation. **Heini Huhtala:** Conceptualization; methodology; software; validation; formal analysis; visualization; writing – review and editing.

## FUNDING INFORMATION

This study was financially partly supported by the Competitive State Research Financing of the Expert Responsibility area of Tampere University Hospital. In addition, the Cancer Foundation Finland, Mary and Georg C Ehrnrooth Foundation and the Finnish Cultural Foundation obtained external funding by awarding a grant, but they did not have any role in designing the study, collecting, analysing, interpreting the data or writing the manuscript.

## CONFLICT OF INTEREST STATEMENT

The authors declare that they have no conflicts of interest.

## ETHICS STATEMENT

Regional Ethics Committee of the Expert Responsibility area of Tampere University Hospital has approved the study and its consent to participate (number R19028). Each study hospital has approved the study by institutional review boards at their unit.

## Supporting information


**Table S1.** Baseline and clinical characteristics with patients, who survived at least 3 months after surgery, according to preoperative frailty status.

**Table S2.** Factors influencing overall survival with patients who survived at least 3 months after surgery (Cox regression analysis).

**Table S3.** Selected preoperative factors influencing survival with patients who survived at least 3 months after colon cancer surgery (Cox Regression analysis).

## Data Availability

The data that support the findings of this study are available on request from the corresponding author. The data are not publicly available due to privacy or ethical restrictions.
