# Supplementary material for: Clinical Frailty Scale predicts overall survival after colon cancer surgery in people aged 80 years and older: A prospective multicentre observational study
Source: Colorectal Dis. 2025 Aug 5;27(8):e70190. doi: 10.1111/codi.70190 (PMC12326052; doi:10.1111/codi.70190)
Supplement: Supplementary file 1 — Table S1. Baseline and clinical characteristics with patients, who survived at least 3 months after surgery, according to preoperative frailty status. [file CODI-27-0-s003.docx]

| **Supplement 1.** Baseline and clinical characteristics with patients, who survived at least three months after surgery, according to preoperative frailty status. | | | | |
| --- | --- | --- | --- | --- |
|  | **CFS 1-2**  n (%) | **CFS 3**  n (%) | **CFS 4**  n (%) | **CFS 5-9**  n (%) |
| Sex |  |  |  |  |
| Female | 28 (52) | 31 (50) | 37 (70) | 38 (66) |
| Male | 26 (48) | 31 (50) | 16 (35) | 20 (34) |
| Age, med years (range) | 84 (80-92) | 83 (80-92) | 85 (80-95) | 86 (80-97) |
| 80-84 | 34 (63) | 44 (71) | 27 (51) | 23 (40) |
| 85-89 | 17 (31) | 13 (21) | 18 (34) | 19 (32) |
| ≥ 90 | 3 (6) | 5 (8) | 8 (15) | 16 (28) |
| BMI, kg/m^2^ |  |  |  |  |
| < 24 | 18 (33) | 20 (32) | 21 (40) | 16 (28) |
| 24-29 | 30 (56) | 27 (44) | 17 (32) | 28 (48) |
| > 29 | 6 (11) | 15 (24) | 15 (28) | 14 (24) |
| Living status |  |  |  |  |
| Home | 54 (100) | 61 (98) | 52 (98) | 56 (97) |
| Nursing home |  | 1 (2) | 1 (2) | 2 (3) |
| Need for support with activities of daily living |  |  |  |  |
| Independent | 44 (82) | 44 (71) | 28 (53) | 5 (9) |
| Outdoors independent | 6 (11) | 5 (8) | 6 (12) | 13 (22) |
| Out and indoors with housework | 4 (7) | 9 (15) | 14 (26) | 23 (40) |
| Out and indoors with basic activities |  | 4 (6) | 5 (9) | 17 (29) |
| Mobility outdoors |  |  |  |  |
| Outdoors unassisted | 51 (94) | 55 (89) | 36 (68) | 21 (36) |
| Outdoors assisted | 3 (6) | 7 (11) | 17 (32) | 29 (50) |
| No outdoor activity |  |  |  | 8 (14) |
| Mobility |  |  |  |  |
| Independent | 52 (96) | 51 (82) | 26 (49) | 9 (16) |
| Independent with walking aid | 2 (4) | 11 (18) | 27 (51) | 41 (71) |
| Dependent of support care or unable to move |  |  |  | 8 (13) |
| Use of walking aid | 9 (17) | 14 (23) | 31 (60) | 51 (88) |
| Number of medications |  |  |  |  |
| < 5 | 29 (54) | 28 (45) | 20 (38) | 9 (16) |
| ≥ 5 | 25 (46) | 34 (55) | 33 (62) | 49 (84) |
| Comorbidities |  |  |  |  |
| Hypertension | 36 (67) | 46 (74) | 35 (67) | 43 (81) |
| Cardiovascular disease^*^ | 23 (43) | 29 (47) | 28 (53) | 43 (74) |
| Diabetes | 10 (19) | 24 (39) | 14 (26)) | 24 (38) |
| Cerebrovascular disease | 3 (6) | 2 (3) | 11 (21) | 13 (22) |
| Pulmonary disease | 5 (9) | 6 (10) | 12 (23) | 5 (9) |
| History of cognitive impairment | 12 (22) | 15 (24) | 17 (32) | 27 (47) |
| G8 score (0-17) |  |  |  |  |
| 0-11 | 14 (26) | 23 (37) | 30 (56) | 38 (66) |
| 12-14 | 28 (52) | 33 (53) | 21 (40) | 18 (31) |
| > 14 | 12 (22) | 6 (10) | 2 (4) | 2 (3) |
| Charlson Comorbidity Index (4-15) |  |  |  |  |
| 4-6 | 44 (81) | 42 (68) | 31 (59) | 24 (41) |
| ≥ 7 | 10 (19) | 20 (32) | 22 (41) | 34 (59) |
| ASA score (2-4) |  |  |  |  |
| 2 | 29 (54) | 19 (31) | 7 (13) | 4 (7) |
| 3 | 25 (46) | 41 (66) | 41 (78) | 45 (78) |
| 4 |  | 2 (3) | 5 (9) | 9 (15) |
| Mini Nutritional Assessment-Short Form (0-14) |  |  |  |  |
| 0-7 (malnutrition) | 4 (7) | 12 (19) | 12 (23) | 21 (36) |
| 8-11 (risk of malnutrition) | 37 (69) | 41 (66) | 40 (75) | 35 (60) |
| ≥12 (normal nutrition) | 13 (24) | 9 (15) | 1 (2) | 2 (4) |
| Haemoglobin (g/L) |  |  |  |  |
| ≤ 120 | 30 (56) | 37 (60) | 41 (77) | 42 (72) |
| > 120 | 24 (44) | 25 (40) | 12 (23) | 16 (28) |
| Albumin (g/L) (missing 19) |  |  |  |  |
| ≤ 30 | 3 (6) | 11 (20) | 9 (17) | 15 (29) |
| 31-34 | 17 (34) | 13 (24) | 20 (39) | 17 (33) |
| > 34 | 30 (60) | 31 (56) | 23 (44) | 19 (38) |
| Estimated glomerular filtration rate (GFR) |  |  |  |  |
| < 45 | 6 (11) | 10 (16) | 12 (23) | 17 (29) |
| 45-60 | 9 (17) | 14 (23) | 14 (26) | 19 (33) |
| > 60 | 39 (72) | 38 (61) | 27 (51) | 22 (38) |
| Procedure |  |  |  |  |
| Right-sided colectomy | 40 (74) | 38 (62) | 36 (68) | 44 (76) |
| Left-sided colectomy | 14 (26) | 22 (35) | 16 (30) | 13 (22) |
| Another colonic resection |  | 2 (3) | 1 (2) | 1 (2) |
| Type of surgery |  |  |  |  |
| Open | 5 (9) | 11 (18) | 9 (17) | 17 (29) |
| Laparoscopy | 49 (91) | 44 (71) | 36 (68) | 35 (61) |
| Conversion |  | 7 (11) | 8 (15) | 6 (10) |
| Postoperative complications | 6 (11) | 28 (45) | 25 (47) | 29 (50) |
| Surgical complications | 5 (9) | 17 (27) | 13 (25) | 13 (28) |
| Non-surgical complications | 2 (4) | 11 (18) | 12 (23) | 19 (33) |
| Clavien Dindo Classification |  |  |  |  |
| 0 | 48 (89) | 34 (55) | 28 (53) | 29 (50) |
| I-II | 5 (9) | 17 (27) | 22 (41) | 20 (34) |
| III-V | 1 (2) | 11 (18) | 3 (6) | 9 (16) |
| Length of hospitalization days (range) | 4 (2-33) | 5 (2-35) | 7 (1-15) | 6 (2-36) |
| Reoperation | 1 (2) | 9 (15) | 1 (2) | 7 (13) |
| Readmission | 4 (7) | 9 (15) | 3 (6) | 5 (9) |
| TNM-stage |  |  |  |  |
| I | 10 (19) | 8 (13) | 24 (45) | 11 (19) |
| II | 28 (52) | 39 (63) | 19 (36) | 27 (47) |
| III | 16 (29) | 15 (24) | 10 (19) | 20 (34) |
| Postoperative adjuvant therapy (Stage III) | 11 (21) | 7 (12) | 1 (2) | 3(5) |

*Coronary Heart Disease + Congestive Heart Failure + Peripheral Artery Disease + Arrhythmia
