# Supplementary material for: Clinical Frailty Scale predicts overall survival after colon cancer surgery in people aged 80 years and older: A prospective multicentre observational study
Source: Colorectal Dis. 2025 Aug 5;27(8):e70190. doi: 10.1111/codi.70190 (PMC12326052; doi:10.1111/codi.70190)
Supplement: Supplementary file 2 — Table S2. Factors influencing overall survival with patients who survived at least 3 months after surgery (Cox regression analysis). [file CODI-27-0-s001.docx]

**Supplement 2** Factors influencing overall survival with patients who survived at least three months after surgery (Cox regression analysis)

|  |  | **Univariate**  **analysis** |  |  |
| --- | --- | --- | --- | --- |
|  | **n** | **HR** | **95% CI** | **p-value** |
| Age, years |  |  |  |  |
| 80-84 | 128 | 1 |  |  |
| 85-89 | 67 | 2.31 | 1.40-3.82 | **0.001** |
| 90- | 32 | 3.16 | 1.73-5.76 | **<0.001** |
| Gender |  |  |  |  |
| Female | 134 | 1 |  |  |
| Male | 93 | 1.01 | 0.64-1.58 | 0.974 |
| BMI, kg/m^2^ |  |  |  |  |
| <24 | 75 | 1 |  |  |
| 24-29 | 102 | 0.73 | 0.44-1.21 | 0.219 |
| >29 | 50 | 0.93 | 0.52-1.67 | 0.806 |
| Type of living |  |  |  |  |
| Home | 223 | 1 |  |  |
| Nursing home | 4 | 1.33 | 0.33-5.42 | 0.694 |
| Need for support with activities in daily living |  |  |  |  |
| Independent | 121 | 1 |  |  |
| Outdoors independent | 30 | 2.53 | 1.35-4.73 | **0.004** |
| Out and indoors with housework | 50 | 2.05 | 1.17-3.62 | **0.013** |
| Out and indoors with basic activities | 26 | 2.62 | 1.38-4.98 | **0.003** |
| Mobility outdoors |  |  |  |  |
| Outdoors unassisted | 163 | 1 |  |  |
| Outdoors assisted | 56 | 1.60 | 0.98-2.62 | 0.060 |
| No outdoor activity | 8 | 4.01 | 1.70-9.44 | **0.001** |
| Mobility |  |  |  |  |
| Independent | 138 | 1 |  |  |
| Independent with walking aid | 81 | 2.18 | 1.37-3.47 | **0.001** |
| Dependent of support care or unable to move | 8 | 4.28 | 1.79-10.2 | **0.001** |
| Hospital admissions <6 months |  |  |  |  |
| None | 117 | 1 |  |  |
| One or more | 110 | 2.39 | 1.50-3.81 | **<0.001** |
| Number of medications |  |  |  |  |
| 0-4 | 86 | 1 |  |  |
| ≥ 5 | 141 | 1.39 | 0.86-2.24 | 0.176 |
| Congestive Heart Disease |  |  |  |  |
| No | 177 | 1 |  |  |
| Yes | 37 | 1.65 | 0.97-2.81 | 0.064 |
| Coronary Artery Disease |  |  |  |  |
| No | 162 | 1 |  |  |
| Yes | 55 | 1.49 | 0.90-2.45 | 0.119 |
| Hypertension |  |  |  |  |
| No | 61 | 1 |  |  |
| Yes | 160 | 1.12 | 0.67-1.88 | 0.672 |
| Chronic obstructive pulmonary disease (COPD) |  |  |  |  |
| No | 213 | 1 |  |  |
| Yes | 9 | 2.10 | 0.85-5.21 | 0.110 |
| Diabetes |  |  |  |  |
| No | 155 | 1 |  |  |
| Yes | 70 | 0.70 | 0.42-1.16 | 0.163 |
| Renal failure |  |  |  |  |
| No | 179 | 1 |  |  |
| Yes | 40 | 1.75 | 1.04-2.95 | **0.035** |
| Cerebral stroke |  |  |  |  |
| No | 187 | 1 |  |  |
| Yes | 29 | 1.53 | 0.84-2.79 | 0.163 |
| Cognition disorders |  |  |  |  |
| No | 156 | 1 |  |  |
| Yes | 71 | 0.96 | 0.59-1.56 | 0.874 |
| G8 score |  |  |  |  |
| 0-11 | 105 | 3.83 | 1.19-12.3 | **0.024** |
| 12-14 | 100 | 2.31 | 0.70-7.57 | 0.167 |
| >14 | 22 | 1 |  |  |
| Clinical Frailty Scale (CFS) |  |  |  |  |
| 1-2 | 54 | 1 |  |  |
| 3 | 62 | 1.73 | 0.82-3.63 | 0.150 |
| 4 | 53 | 1.73 | 0.79-3.80 | 0.167 |
| 5-9 | 58 | 4.05 | 2.04-8.04 | **<0.001** |
| Charlson Comorbidity Index (CCI) |  |  |  |  |
| 4-6 | 140 | 1 |  |  |
| >6 | 86 | 1.38 | 0.89-2.17 | 0.155 |
| ASA score |  |  |  |  |
| 2 | 59 | 1 |  |  |
| 3 | 149 | 1.40 | 0.80-2.46 | 0.244 |
| 4 | 16 | 2.94 | 1.36-6.36 | **0.006** |
| Mini-Nutritional Assessment-Short Form (MNA-SF) |  |  |  |  |
| 0-7 | 49 | 3.17 | 1.31-7.69 | **0.011** |
| 8-11 | 153 | 1.25 | 0.53-2.92 | 0.611 |
| >11 | 25 | 1 |  |  |
| Haemoglobin (g/l) |  |  |  |  |
| ≤ 120 | 150 | 2.42 | 1.40-4.20 | **0.002** |
| > 120 | 77 | 1 |  |  |
| Albumin (g/l) |  |  |  |  |
| <31 | 38 | 2.98 | 1.68-5.29 | **<0.001** |
| 31-34 | 67 | 1.29 | 0.74-2.27 | 0.375 |
| >34 | 103 | 1 |  |  |
| Estimated glomerular filtration rate GFR (ml/min) |  |  |  |  |
| <45 | 45 | 2.11 | 1.23-3.61 | **0.006** |
| 45-60 | 56 | 1.63 | 0.96-2.80 | 0.073 |
| >60 | 126 | 1 |  |  |
| Type of operation |  |  |  |  |
| Laparoscopy | 164 | 1 |  |  |
| Open | 42 | 1.73 | 1.03-2.91 | **0.039** |
| Conversion | 21 | 1.38 | 0.66-2.93 | 0.394 |
| Surgical complication |  |  |  |  |
| No | 179 | 1 |  |  |
| Yes | 48 | 1.13 | 0.67-1.92 | 0.643 |
| Non-surgical complication |  |  |  |  |
| No | 183 |  |  |  |
| Yes | 44 | 1.95 | 1.18-3.20 | **0.009** |
| Clavien Dindo classification |  |  |  |  |
| 0 | 139 | 1 |  |  |
| I-II | 64 | 1.48 | 0.88-2.47 | 0.137 |
| III-V | 24 | 2.32 | 1.23-4.36 | **0.009** |
| Tumour stage |  |  |  |  |
| I | 53 | 1 |  |  |
| II | 113 | 1.33 | 0.67-2.63 | 0.419 |
| III | 61 | 3.41 | 1.73-6.74 | **<0.001** |
| Postoperative adjuvant therapy (Stage III) |  |  |  |  |
| No | 205 | 1 |  |  |
| Yes | 22 | 1.22 | 0.59-2.53 | 0.601 |
