# Supplementary material for: Clinical Frailty Scale predicts overall survival after colon cancer surgery in people aged 80 years and older: A prospective multicentre observational study
Source: Colorectal Dis. 2025 Aug 5;27(8):e70190. doi: 10.1111/codi.70190 (PMC12326052; doi:10.1111/codi.70190)
Supplement: Supplementary file 3 — Table S3. Selected preoperative factors influencing survival with patients who survived at least 3 months after colon cancer surgery (Cox Regression analysis). [file CODI-27-0-s002.docx]

**Supplement 3** Selected preoperative factors influencing survival with patients who survived at least three months after colon cancer surgery (Cox Regression analysis)

|  | **Univariate** |  |  | **Multivariable** |  |  |
| --- | --- | --- | --- | --- | --- | --- |
|  | **HR** | **95% CI** | **p-value** | **HR** | **95% CI** | **p-value** |
| ASA |  |  |  |  |  |  |
| 2 | 1 |  |  | 1 |  |  |
| 3 | 1.40 | 0.80-2.46 | 0.244 | 0.74 | 0.37-1.51 | 0.412 |
| 4 | 2.94 | 1.36-6.36 | **0.006** | 1.45 | 0.50-4.19 | 0.498 |
| AA-CCI |  |  |  |  |  |  |
| 4–6 | 1 |  |  | 1 |  |  |
| >6 | 1.38 | 0.89–2.17 | 0.155 | 0.86 | 0.49–1.54 | 0.621 |
| CFS |  |  |  |  |  |  |
| 1–2 | 1 |  |  | 1 |  |  |
| 3 | 1.73 | 0.82–3.63 | 0.150 | 1.26 | 0.54–2.95 | 0.599 |
| 4 | 1.73 | 0.79–3.80 | 0.167 | 1.24 | 0.50–3.06 | 0.647 |
| 5–9 | 4.05 | 2.04–8.04 | **<0.001** | 2.94 | 1.23–7.03 | **0.015** |
| G8 score |  |  |  |  |  |  |
| 0–11 | 3.83 | 1.19-12.3 | **0.024** | 2.01 | 0.59–6.92 | 0.266 |
| 12–14 | 2.31 | 0.70–7.57 | 0.167 | 1.45 | 0.42–5.04 | 0.562 |
| >14 | 1 |  |  | 1 |  |  |
| Haemoglobin (g/l) |  |  |  |  |  |  |
| ≤120 | 2.42 | 1.40–4.20 | **0.002** | 1.82 | 0.97–3.39 | 0.061 |
| >120 | 1 |  |  | 1 |  |  |
| Albumin (g/l) |  |  |  |  |  |  |
| <31 | 2.98 | 1.68–5.29 | **<0.001** | 1.97 | 1.03–3.74 | **0.039** |
| 31–34 | 1.29 | 0.74–2.27 | 0.375 | 1.00 | 0.55–1.81 | 0.998 |
| >34 | 1 |  |  | 1 |  |  |
| GFR (ml/min) |  |  |  |  |  |  |
| <45 | 2.11 | 1.23–3.61 | **0.006** | 2.02 | 1.07–3.82 | **0.030** |
| 45–60 | 1.63 | 0.96–2.80 | 0.073 | 1.54 | 0.84–2.82 | 0.160 |
| >60 | 1 |  |  | 1 |  |  |

Abbreviations: ASA, American Society of Anaesthesiologists; AA-CCI, Age-Adjusted Charlson Comorbidity Index; CFS, Clinical Frailty Scale; GFR, Estimated glomerular filtration rate
